# Case Report: A novel t(15;17)(q24;q11.2) translocation involving *NF1::SCAMP5* fusion in a patient with myeloproliferative neoplasms

**DOI:** 10.3389/fonc.2025.1600963

**Published:** 2025-07-21

**Authors:** Yuying Chang, Yakun Chen, Weiwei Zhao, Guomin Shen, Sujuan Guo, Wei Wang

**Affiliations:** ^1^ Department of Hematology, The 2nd Affiliated Hospital of Harbin Medical University, Harbin, China; ^2^ Beijing Diagnostic Center, Department of Hematology of DiAn Diagnostic, Beijing, China; ^3^ Department of Cell Biology, School of Basic Medicine Harbin Medical University, Harbin, China

**Keywords:** myeloproliferative neoplasms, t(15, 17), NF1::SCAMP5, primary myelofibrosis, novel fusion

## Abstract

Myeloproliferative neoplasms (MPNs) are a heterogeneous group of disorders characterized by the abnormal proliferation of terminally differentiated myeloid cells. While cytogenetic abnormalities such as t(15;17) are documented in MPNs, the specific translocation resulting in NF1::SCAMP5 fusion has not been previously reported. Here we present a 69-year-old female patient with anemia and splenomegaly, exhibiting CALR exon 9 mutation (c.1099_1150del52) and JAK2 V617F negativity. Cytogenetic analysis revealed t(15;17)(q24;q11.2), distinct from the classical APL-associated t(15;17)(q22;q21), with RNA-Seq confirming a novel NF1::SCAMP5 fusion. Bone marrow biopsy showed MF-1 fibrosis and megakaryocyte depletion, deviating from typical primary myelofibrosis histology. The patient achieved stable disease post-ruxolitinib treatment. This case highlights a unique molecular-pathological profile, suggesting NF1::SCAMP5 may define a provisional MPN subtype with distinct genetic features, warranting further study to elucidate its clinical significance.

## Introduction

Myeloproliferative neoplasms (MPNs) are a group of indolent hematopoietic disorders, encompassing essential thrombocytosis, polycythemia vera, and primary myelofibrosis (PMF) (1). The concept of myeloproliferative disorders was created by William Dameshek, who also conceptualized the term “myeloproliferative neoplasms (MPN)” in 1951 (2, 3). There are several symptoms of PMF, including leucoerythroblastic, organomegaly, extramedullary hematopoiesis, and bone marrow fibrosis (4). It is a type of *BCR-ABL*-negative MPN (4). The JAK-STAT pathway is activated in about 90% of MPN cases, often accompanied by somatic mutation in the *CALR* and *MPL* genes (5, 6). Cytogenetic abnormalities (CA) are also observed in some of MPN cases, such as trisomy 1q, del(7)(q21-36), trisomy 8, trisomy 9, del(13)(q13-q21), del(17)(p11-13), i(17q), del(20)(q11.2q13.1), and t(15;17) (7, 8). However, the t(15;17) translocation resulting in the NF1::SCAMP5 fusion has not been reported in previous cases.

We report a rare case presenting with anemia, splenomegaly, and increased basophil. The RT-PCR analysis failed to detect the *BCR-ABL* fusion gene and did not identify any mutations in the *MPL* gene or the *JAK2* gene, including the *JAK2* V617F site. Additionally, the *CALR* gene exon 9 has a mutation, specifically *CALR* c.1099_1150del52(p.L367fs*46), and t(15;17) (q24,q11.2) was identified by cytogenetics analysis. It is different from the classical t(15;17)(q22,q21) in APL. The breakpoint is at *NF1* gene and *SCAMP5* gene which is different from the classical t(15;17(q22,q21), leading to the formation of a *NF1::SCAMP5* fusion gene. The fusion gene has not been reported in MPN cases, and its clinical significance remains unclear. We identified a novel chromosomal translocation in MPN cases, offering a new angle to advance MPN research further.

**Figure 1:**
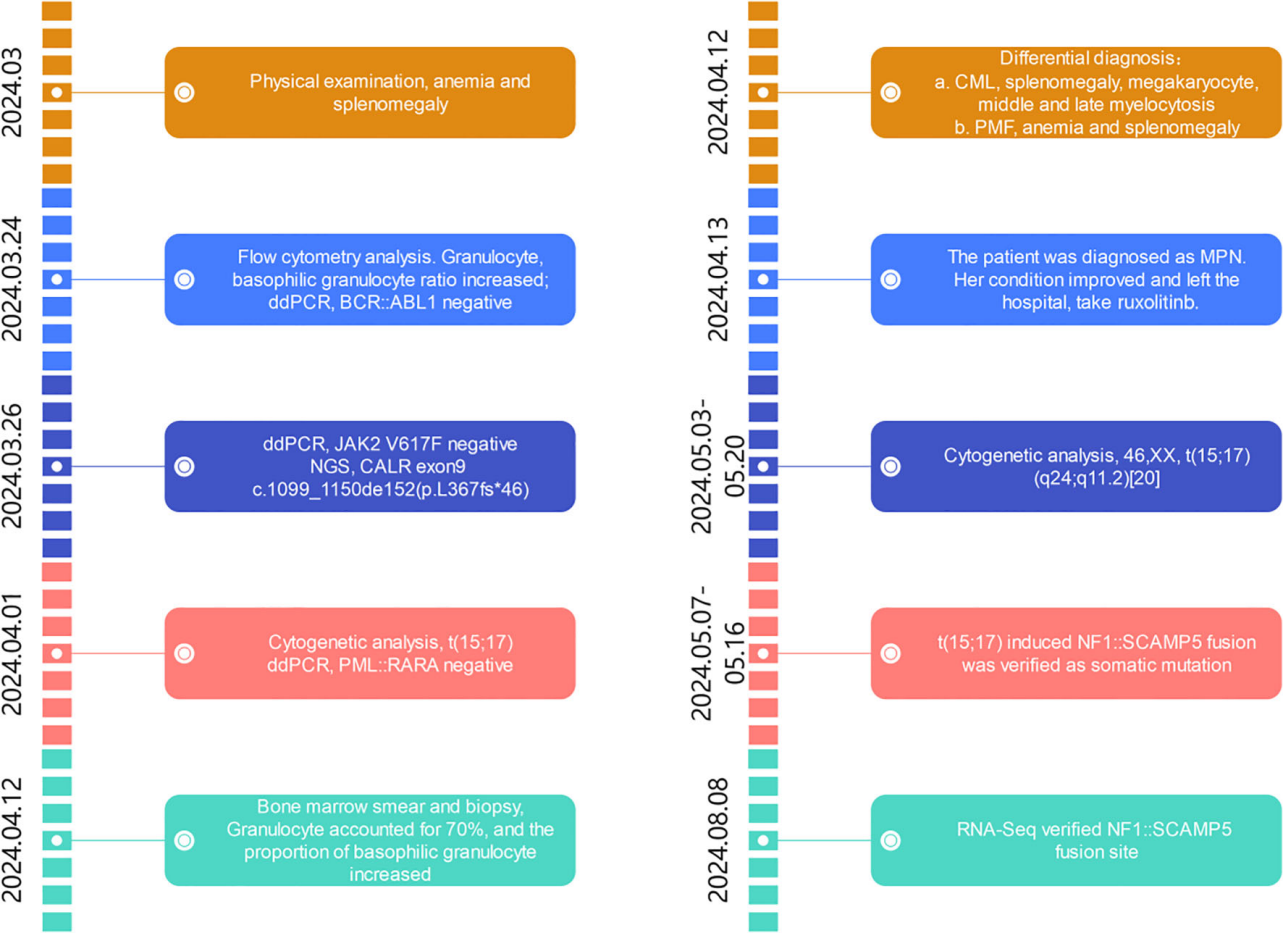
Timeline of events from first presentation to 6-month follow-up. ddPCR, digital droplet PCR; NGS, next-generation sequencing; CALR, calreticulin gene; CML, chronic myelogeous leukemia; PMF, primary myelofibrosis; MPN, myeloproliferative neoplasm.

A summary of events is shown in **Figure 1**, from the patient’s first presentation to the 6-month follow-up.

## Case description

A 69-year-old female patient was initially admitted to the hospital on the grounds of anemia. She had no obvious cause for physical examination and was diagnosed with mild anemia and an enlarged spleen. On detailed questioning, she went to the hospital for further examination. She reported no history of infection, surgery, allergy, or blood transfusion history. In addition, she has been suffering from Parkinson’s disease for more than 10 years, taking Antan, amantadine, midoban, and other drugs.

The physical examination was remarkable for splenomegaly, with 7 cm as the distance from the left midclacicular line and left costal margin to the inferior margin of the spleen, 5 cm from the intersection of left midclavicular line and left costal margin to the farthest point the spleen, and 0 cm from the right margin of the spleen to the anterior median line. No significant enlargement of cervical, axillary, or superficial inguinal lymph nodes was detected.

The peripheral blood complete blood count revealed that the white blood cell (WBC) was 6.9 × 10^9^/L with an increased basophilic granulocyte count (BASO), which was 0.16 × 10^9^/L. Hemoglobin was at 108 g/L, and the platelet count was at 133 × 10^9^/L. The platelet aggregation function was normal.

The sinus rhythm exhibited a normal cardiac axis. The lung CT showed interstitial changes in both lungs: bilateral pulmonary maculata, bilateral localized emphysema, and bilateral localized pleural hypertrophy. The patient was also found to have an enlarged cardiac shadow and slightly thickened pericardium. The three-dimensional ultrasound indicated mild diffuse liver changes, chronic cholecystitis, and splenomegaly.

**Figure 2:**
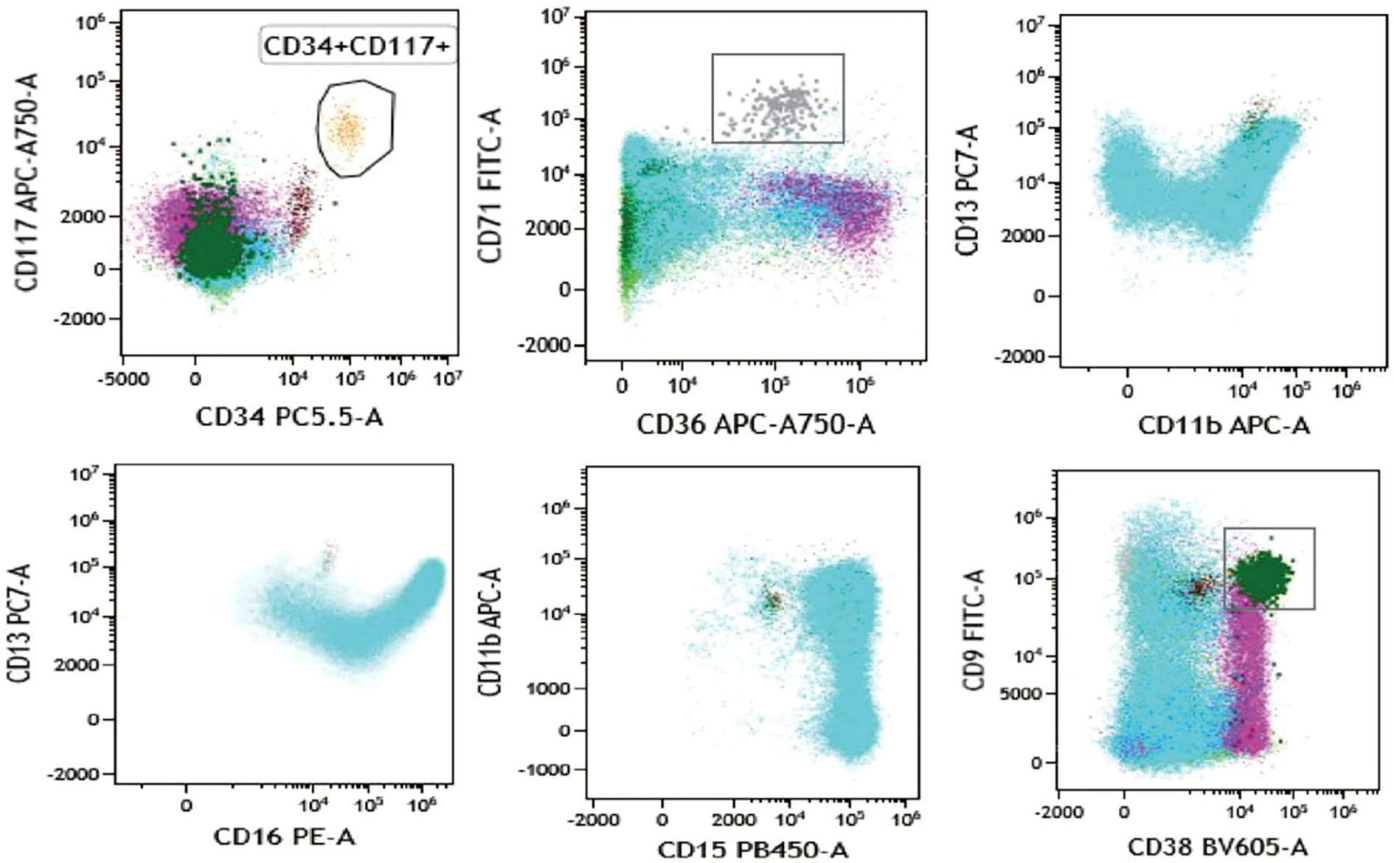
Flow cytometry analysis showing the biomarkers’ expression of bone marrow sample. CD34+ CD117+ cells are presented in aurantium color. CD36+ CD71+ cells indicate nucleated red cells and are presented in gray color. CD13, CD15, CD16, and CD11b indicate granulocyte and are presented in blue color. CD9+ CD38+ cells indicate basophil and are presented in dark green color.

The flow cytometry analysis showed the increased proportion of granulocytes (79.40% of nucleated cells) and basophilic granulocytes (1.28% of nucleated cells), and the phenotype of cells is normal (**Figure 2**; refer to **Table 1** for antibody panel details). Although the proportion of nucleated red blood cells decreased (0.80% of nucleated cells), the cells also show a normal phenotype (**Figure 2**). In the initial diagnosis, chronic myelogenous leukemia (CML) and PMF were considered. These two types of leukemia all show enlarged spleen. Thus, bone marrow biopsy and cytogenetic analysis are needed. To explore the possibility of chronic myeloid leukemia, *BCR::ABL1*(p190/p210/p230) fusion genes were tested and showed negative results.

**Table 1:** Fluorescent channel and antibodies.

KO525	BV605	BV650	PE	FITC	PC5.5	APC-A750	PC7	APC-A700
CD45	CD19 CD38	CD20	cLambda CD10 CD56 CD16	cKappa CD9 CD71	CD5 CD34 CD64	CD3 CD117 CD36	CD8 CD13	CD4 CD7 CD14
APC	ECD	PB450	BV785					
CD34 CD33 CD11b	CD22 HLA-DR	CD15 CD19	CD138					

**Figure 3:**
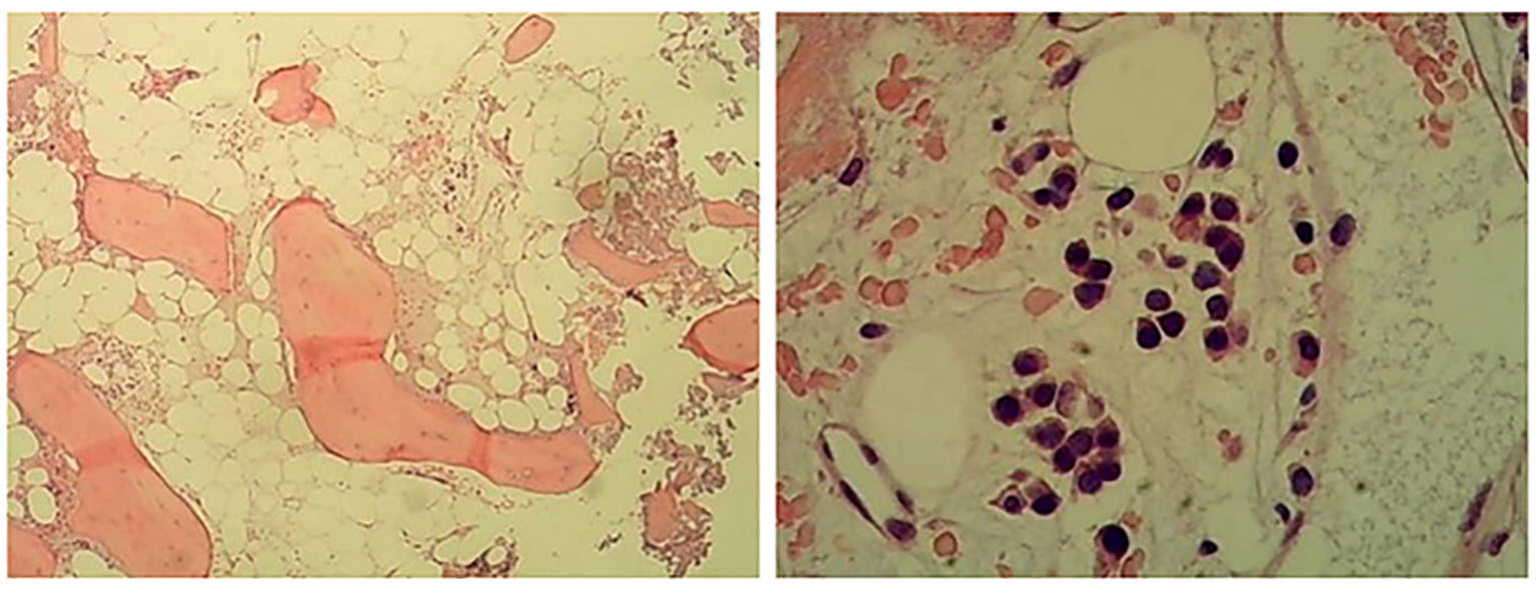
Hematoxylin and eosin (H&E) staining (left) and periodic acid–Schiff (PAS) staining (right) of bone marrow biopsy sample. Hyperplasia was low in most areas of the bone marrow (<5%), and a small number of partial mature granulocytes were scattered or clustered. Megakaryocytes are rare, and megakaryocytes with large cell bodies can be seen. A few small lymphocytes and plasma cells were scattered.

The results of bone marrow aspiration with morphologic analysis demonstrated hypocellular marrow (grade III) with markedly increased granulopoiesis (70%, primarily myelocytes and mature forms) alongside severe erythroid hypoplasia (1%, M:E ratio = 70:1), accompanied by characteristic findings including teardrop erythrocytes, absent megakaryocytes (though platelets were present), and mild monocytosis (9.5%). A subsequent bone marrow biopsy with immunohistochemistry revealed basophilia, confirming hypocellularity (<5% cellularity) in most marrow regions, showing scattered clusters of partially mature granulocytes along with rare but notably enlarged megakaryocytes. The marrow also contained scattered small lymphocytes and plasma cells, while reticulin fiber staining established MF-1 grade fibrosis (**Figure 3**). This combined evaluation suggests a myeloproliferative process with associated fibrotic changes.

The results of immunohistochemistry (IHC) staining showed the following immunophenotypes: CD34 -, CD117 mast cells +, MPO granulocyte cells +, lysozyme part +, CD71 erythroid cells +, CD42b megakarocytes +, CD20 individual +, a small number of CD3 +, CD56 individual +, and CD138 plasma cells +. The results indicated hypohematopoietic function of the bone marrow. It is necessary to detect *JAK2* V617F for further diagnosis. qPCR and Sanger sequencing had not detected *JAK2* V617F mutation, exon 12 mutation, and *PML* exon 10 mutation. *CALR* exon 9 shows a deletion at *CALR* c. 1099_1150del52 (p.L367fs*46). *CALR* exon 9 mutation is a significant indicator of MPN (9).

**Figure 4:**
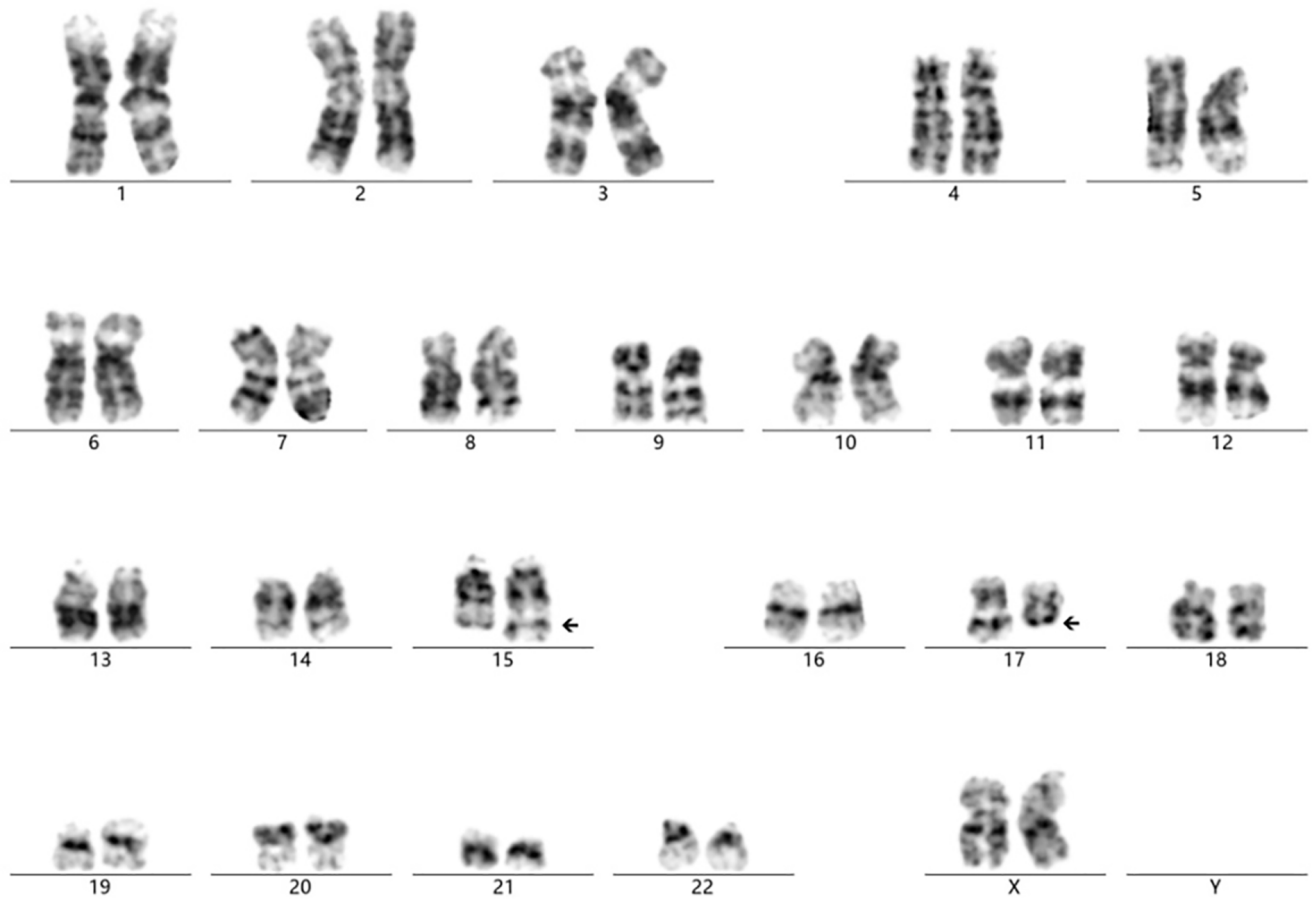
Cytogenetic analysis showing the chromosome translocation of chromosome 15 and chromosome 17. The abnormal karyotype was t(15; 17)(q24; q11.2), as the arrows presented.

**Figure 5:**
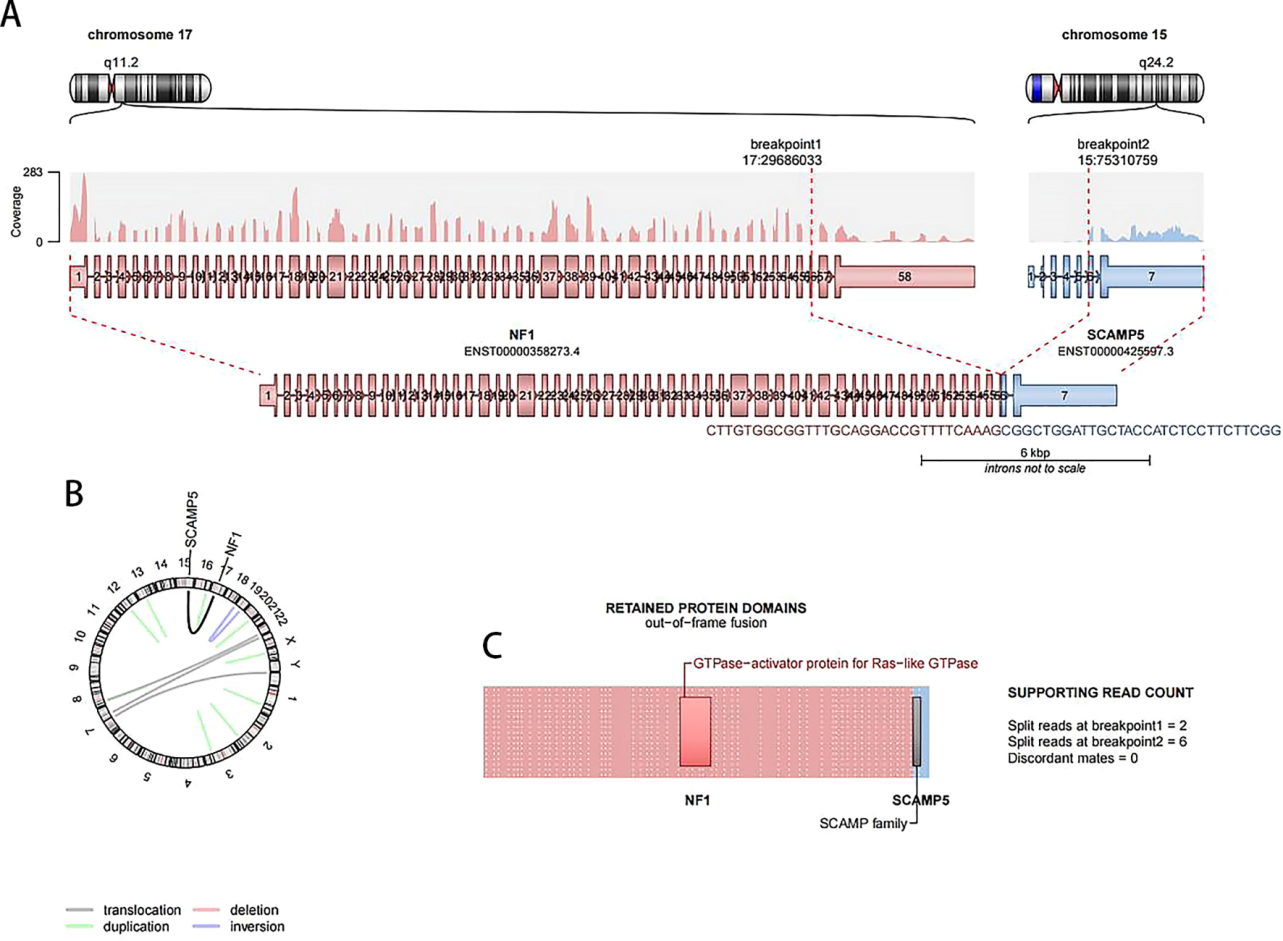
RNA-Seq results showing t(15;17) translocation in the patient’s bone marrow cells. **(A)** The breakpoint in chromosome 15 and 17, *NF1::SCAMP5* fusion site, is presented. **(B)** *NF1::SCAMP5* fusion gene is presented in circle plot. **(C)** Out-of-frame fusion protein schematic drawing.

**Figure 6:**
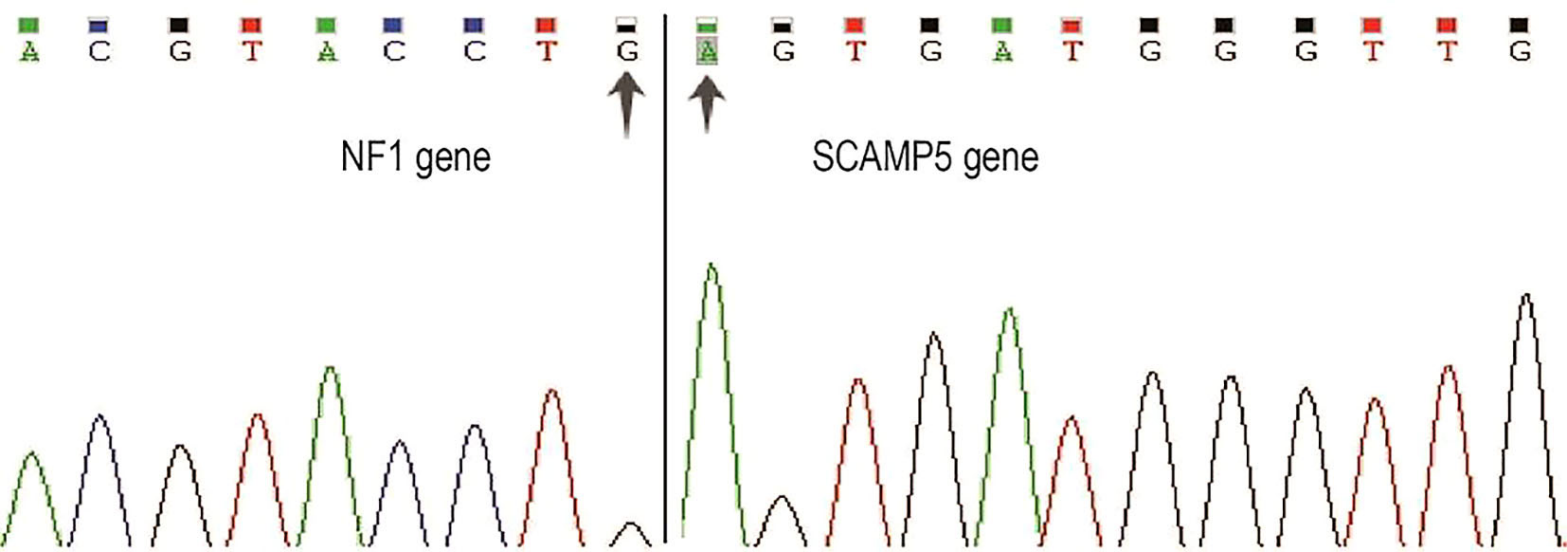
Sanger sequencing of *NF1::SCAMP5* fusion gene. Primers were designed based on RNA-Seq results. PCR products were purified using the same primers for sanger sequencing. Two arrows indicate NF1 and SCAMP5 genes’ fusion location.

The cytogenetic analysis revealed the t(15;17)(q24;q11.2) translocation in all 20 examined metaphase cells, described as 46,XX,t(15;17)(q24;q11.2) [20] (**Figure 4**). The breakpoints were located at chromosome 15 and 17, but the *PML::RARA* fusion gene was not found. It indicates that the translocation is not the classical t(15;17) translocation in acute promyelocytic leukemia (APL). RNA-Seq was performed with HiSeq PE150 to verify the translocation breakpoint in the two chromosomes, showing the breakpoint regions as exon 6 of chromosome 15 in SCAMP5 gene and exon 56 of chromosome 17 in *NF1* gene. It resulted in a *NF1::SCAMP5* fusion gene (**Figure 5A**). The chromosome circle plot shows t(15;17) translocation as well (**Figure 5B**). The retained protein domains is made up of *NF1* and *SCAMP5* genes (**Figure 5C**). The germ line test ruled out inherited variants, and Sanger sequencing further confirmed the fusion of *NF1* and *SCAMP5* genes (**Figure 6**).

In prior studies, it was not reported that the *NF1::SCAMP5* fusion gene exists. The clinical significance remains unclear. *NF1* gene is a tumor suppressor gene, and its deficiency occurs in 5% of myeloid malignancies (10). It is located at 17q11.2 and encodes the RAS-GTPase-activating protein neurofibromin. Its mutation affects the RAS-MAPK pathway (11). *NF1* mutations were accompanied by other genes, such as *NPM1*, *WT1*, *TP53*, and others, and these are associated with poor prognosis in young AML patients (11). *SCAMP5* belongs to SCAMP family, which is widely expressed by neurons and non-neurons in various tissues and cells. It can enhance the activity of calcium-regulated signal peptide-containing cytokines, but its prognostic significance in AML remains unclear (12).

Given the molecular findings (CALR mutation + NF1::SCAMP5 fusion) and MF-1 fibrosis, the patient was provisionally classified as “MPN with features overlapping early-stage PMF”, though the megakaryocyte-poor histology suggests a potential novel subtype requiring further validation. The patient was diagnosed on April 13, 2024 and initiated on ruxolitinib treatment. She subsequently returned to her local hospital where follow-up blood tests were performed on May 7, 2024. The peripheral blood complete blood count was demonstrated as follows: white blood cell count was 8.23 × 10^9^/L (normal range), hemoglobin was at 120 g/L (returned to normal levels), and the platelet count was 121 × 10^9^/L. While the hematologic parameters showed improvement (particularly hemoglobin normalization), an abdominal ultrasound for spleen measurement was not performed during this follow-up. Unfortunately, subsequent treatment outcomes and monitoring data beyond May 2024 were not available for continued evaluation.

## Discussion

This report presents a novel case of MPN characterized by translocation t(15;17)(q24;q11.2), which has not been previously documented in the literature. The patient initially presented with anemia and splenomegaly during physical examination, leading to hospitalization for further evaluation of anemia. The bone marrow cytology revealed no abnormal blast cells, with only a small population of teardrop erythrocytes observed. The flow cytometry analysis showed no evidence of abnormal immunophenotypic cells, while the chromosomal karyotyping demonstrated a 46,XX,t(15;17) pattern. Regarding diagnostic considerations, although the bone marrow biopsy indicated neutrophilia, the absence of CML-specific karyotypes and *BCR-ABL* fusion gene excluded the diagnosis of myeloid leukemia. Furthermore, while the t(15;17) translocation is typically associated with acute promyelocytic leukemia APL, the *PML::RARA* fusion gene was negative in this case. The clinical manifestations and bone marrow cytology findings were also inconsistent with the APL diagnosis.

Notably, both of our diagnostic evaluations (bone marrow aspiration with morphologic analysis and bone marrow biopsy supplemented by immunohistochemistry) showed rare megakaryocytes, which contrasts with the typical megakaryocytic proliferation required by the WHO 2022 classification of hematopoietic tumors for overt PMF (13). While megakaryocyte depletion is atypical for classical PMF, recent studies suggest that specific molecular subtypes (e.g., CALR ins5 mutations) may exhibit reduced megakaryocyte proliferation despite thrombocytosis, as seen in murine models where ins5 primarily increases megakaryocyte ploidy rather than numbers (14). Additionally, CALR del52 homozygous mice develop BM hypocellularity with splenic extramedullary hematopoiesis, mirroring the “early-stage PMF” phenotype proposed by the WHO 2022 criteria. The presence of MF-1 fibrosis, CALR mutation, and t(15;17)-driven NF1::SCAMP5 fusion may represent an atypical molecular–pathological profile warranting reclassification as “provisional MPN with unique genetic features” until further cases are reported.

The t(15;17) translocation is most commonly identified in APL. This translocation typically occurs at t(15;17)(q22;q12-21), resulting in the formation of the *PML::RARA* fusion gene. The fusion oncoprotein produced by this genetic alteration can induce leukemogenesis by disrupting normal myeloid lineage development (15). In rare cases, rearrangements at 17q21 lead to the fusion of the *RARA* gene with alternative partners, such as *NPM*, *PLZF*, and *NuMA* (16). These genetic rearrangements facilitate the formation of homodimers between *RARA* and its partner genes, ultimately disrupting the RARA signaling pathways (17). Consequently, these fusion events contribute to the development of leukemia.

Further experimental investigations were conducted following the MPN diagnosis. The results revealed a novel association between the *NF1* gene (located at 17q11.2) and the *SCAMP5* gene (located at 15q24.2). Genomic cleavage and recombination events between these loci led to the formation of the *NF1::SCAMP5* fusion gene. To our knowledge, this is the first report on the *NF1::SCAMP5* fusion, as no prior studies have documented its existence or functional implications.

### Cytogenetic abnormalities and t(15;17) translocation

Philadelphia chromosome-negative MPNs are characterized by three principal driver mutations in clinical studies: *JAK2*, *MPL*, and *CALR* mutations. All three genetic alterations converge on the constitutive activation of the JAK-STAT pathway (18). This aberrant signaling subsequently triggers the downstream activation of the PI3K-AKT, JAK-STAT, and RAF-MEK-ERK cascades, ultimately promoting the transcriptional upregulation of genes involved in pathological cell proliferation—a hallmark of polycythemia vera, essential thrombocythemia, and PMF (19). While chromosomal translocations are rare in MPNs, we identified a novel *NF1::SCAMP5* fusion gene resulting from a t(15;17)(q24.2;q11.2) translocation in our case.

The *NF1* gene mutation can induce a familial cancer syndrome which is called neurofibromatosis type1 (NF1) (20). Children with *NF1* mutation would be predisposed to juvenile myemonocytic leukemia (JMML), which is a type of aggressive MPN. As *NF1* is a tumor suppressor gene, it is a GTPase-activating protein (GAP) which negatively regulate Ras signaling. The loss of it in mice hematopoietic systems would induce MPN (20). The somatic inactivation of *NF1* could lead to myelo-erythroid progenitors’ and committed cells’ proliferation and expansion (21). *NF1*-mutated JMML presented abnormal Raf/MEK/ERK signaling (22). The MEK inhibitor PD0325901 treatment in Mx1-Cre;Nf1flox/flox(Nf1) mice shows reduced myeloproliferation and enhanced erythropoiesis (21).

In addition, a series of genes in the Ras pathway, including *NF1*, *NRAS*, *KRANS*, *PTPN11*, and *CBL*, are presented as a molecular diagnosis in 85% JMML patients (23–27). t(11;17) is identified in one JMML patient, and the *NF1* locus has been disrupted. However, fusion RNA cannot be detected by RNA-Seq, which indicates that the breakpoint is at the intergenic region (28).

Secretory carrier-associated membrane proteins (SCAMPs) are related with multiple human cancers, such as leukemia and pancreatic adenocarcinoma (29). It is a family of transcription factors, including *SCAMP1–5* (30). It participates and control cell–cell adhesion and cancer invasion (31). *SCAMPs* gene mutation is only been detected in below 1% of AML patient. The mRNA levels of *SCAMP2/5* are significantly higher in AML than control patients. *SCAMP5* is highly expressed in bladder cancer, breast cancer, esophageal cancer, lung cancer, melanoma, and leukemia. The high expression of *SCAMP1-5* is correlated with patients’ worse survival (32). The KEGG analysis shows that *SCAMP5* is correlated with the chemokine signaling pathway and cell adhesion molecules (CAMs) (31), so the gene plays an oncogene role in cancer, and it may be used as a diagnostic identifier in cancer.

In our case, the *NF1::SCAMP5* fusion gene was detected by RNA-Seq. It is presented that one to 56 exons in the *NF1* gene fused to six to seven exons of *SCAMP5*. A part of the tumor suppressor fused to parts of the oncogenetic transcription factor exons, leading to leukemia development. As *NF1* is a GTPase-activating protein (GAP) which negatively regulates Ras signaling, the separation of exons 57 and 58 from other exons may lead to the destruction of GTPase-activating function and Ras signaling activation. In addition, exon 5 and exon 6 breakage in the *SCAMP5* gene may also affect the normal structure of transmembrane domain and exocytosis and endocytosis (32).

The hypothesis on the mechanisms of fusion gene promoting cancer progression may be correlated with how tumor suppressor genes are inactivated and oncogenetic transcription factors are over-activated. The *NF1::SCAMP5* fusion gene may be a new type of diagnosis marker of MPN.

## Conclusions

This study reports the first case of t(15;17)(q24;q11.2) leading to NF1::SCAMP5 fusion in an MPN patient with CALR mutation and MF-1 fibrosis. The absence of megakaryocytic proliferation challenges classical PMF criteria, proposing an atypical molecular subtype. The NF1::SCAMP5 fusion may disrupt the NF1 tumor–suppressor function while activating SCAMP5-mediated oncogenic pathways, driving myeloid proliferation. These findings underscore the need to expand the MPN classification to include rare genetic variants and emphasize the role of comprehensive molecular profiling in diagnosis. Further studies are required to validate NF1::SCAMP5 as a diagnostic marker and therapeutic target in MPNs.

## Data Availability

The original contributions presented in the study are included in the article/Supplementary Material. Further inquiries can be directed to the corresponding authors.
